# Does overconfident CEO lead to corporate environmental misconducts? Evidence from China

**DOI:** 10.1371/journal.pone.0309957

**Published:** 2024-09-06

**Authors:** Lu Zhang, Dayuan Li, Zhaohua Xiao, Jialin Jiang, Fenghua Lu

**Affiliations:** 1 School of Business, Hunan First Normal University, Changsha, China; 2 School of Business, Central South University, Changsha, China; 3 School of Management, Hunan University Of Technology and Business, Changsha, China; 4 Business School, Hunan University of Finance and Economics, Changsha, China; OP Jindal Global University, INDIA

## Abstract

Enterprises are drawing growing criticism for violating environmental rules. The research examines whether and how top executives’ mental bias leads to corporate environmental misconduct (CEI). Drawing on upper echelon theory (UET) and agency theory, we link CEO overconfidence with CEI, and explore the boundary conditions from the perspective of management discretion at the governance level. Using a data set covering the Chinese listed enterprises from 2004 to 2016, the empirical results demonstrate that CEO overconfidence positively and markedly influenced CEI. Moreover, shareholder concentration and CEO duality reinforce the relationship between overconfidence and CEI, whereas board independence is the opposite. The findings clarify ecological outcomes of CEO overconfidence and have remarkable significance in theory and practice.

## Introduction

Environmental deterioration and man-made natural disasters have presented a worldwide challenge over the recent decades [1,2]. Firms are regarded as dominant producers of such challenges: the business world often witnesses corporate environmental misconduct [3], such as the emission scandal involving Volkswagen and the decades-long cheating on fuel tests of Mitsubishi Motors. These phenomena raise the following question: Why do firms engage in environmentally irresponsible activities?

Growing bodies of literature have explored contextual and organizational factors that considerably affect corporate environmental conduct and misconduct [4,5]. In terms of contextual factors, existing literature mainly explores the impact of formal institutional environments like environmental regulations [6], environmental tax [7,8], and government subsidies [9,10], alongside informal institutional environments such as media coverage [11] and public attention [12,13] on corporate environmental behavior. In terms of organizational factors, environmental innovation, as a typical corporate environmental conduct, is influenced by different types of corporate culture, such as clan culture and adhocracy culture, which promote corporate environmental innovation, while hierarchy culture is the opposite [14]. Furthermore, Fernandes, Kuzey [15] assert that board diversity, particularly in terms of gender, can lead to more socially responsible decision-making and sustainable practices. Additionally, Zhang, Gong [16] conducted empirical analyses on the relationship between the top management team’s (TMT) regulatory focus and firm environmental misconduct (FEM), concluding that TMTs high in promotion focus are more likely to engage in FEM, whereas TMTs high in prevention focus are less likely to misconduct. Regardless, studies on the role of CEOs remain relatively underdeveloped [17]. Upper echelons theory (UET) believes that the leader’s characteristics significantly affect a company’s strategic choices [18].

Recent research has recently evaluated the impact of CEO’s cognitive and psychological characteristics on a company’s decision-making [19]. CEO overconfidence is one of the key psychological traits, which profoundly affect a sequence of company’s decisions [20], such as enterprise investment [21], corporate social responsibility [22], and corporate risk-taking [23]. In brief, the research on the outcomes of CEO overconfidence has still largely remained on the economic outcomes but rarely extends to the ecological effect [24]. The research aims to elucidate the link between CEO overconfidence and corporate environmental misconduct.

Moreover, if overconfident CEOs indeed affect corporate environmental misconduct, certain conditions influencing the emergence of such a relationship need to be determined. UET has identified managerial discretion, the freedom of action that managers can take [25], as an important moderator. Managerial discretion may be the reason why managers exert more influence over their firms in some situations than in others [26], and corporate governance is an important mechanism to monitor such discretion. Agency theory describes various problems among shareholders and managers [27], and believes that shareholder concentration [28] and the formal and informal powers of independent boards [29] can effectively regulate managers. Thus, the present study investigates the boundary conditions for factors influencing managerial discretion at the governance level that may mitigate or strengthen the overconfidence–environmental misconduct relationship.

Our research has several significant contributions. First, we contribute to the UET—managerial biases and discretion in particular—by extending CEO overconfidence and managerial discretion research into the ecological context, as the existing research primarily emphasizes economic outcomes. Second, we are devoted to studying corporate social responsibility (CSR) by explicitly analyzing the less discussed corporate environmental misconduct from the UET perspective [30]. Recent research advocates testing the role of CEOs in corporate misconduct [31]. We respond to this call through studying the effect of CEO overconfidence on corporate participation in environmental misconduct. Third, we prove the managers’ psychological biases and their ecological outcomes in China, a collectivist society with severe environmental problems different from the more individualist and less ecologically-concerned Western contexts of prior studies [32].

The rest of this article states as follows. The theoretical background of CEO overconfidence is presented in the next section. Subsequently, there are research hypotheses, research methods, empirical results and robustness testing, as well as discussions and conclusions on the study.

## Theoretical background

Corporate environmental misconduct is widely regarded as an illegal act that endangers the public, violates the expected standards of environmental behavior, and generally poses a threat to human health or environmental stability [33]. With the increasingly severe environmental deterioration and enhancement of public environmental awareness, corporate environmental conduct and misconduct have become an increasingly important research and managerial topic [34]. The question of what leads to corporate environmental misconduct has prompted heated discussions. One of the most prevailing views is the environmental determinism of external systems [35]. For instance, it’s demonstrated that institutional and social norms affect corporate adoption of constructive or destructive activities concerning environmental issues by Bowen and De Clercq [36]. However, corporate environmental behavior is not only a response to outside force, but also to inside factors, including managerial structures, research and development expenditure [17], particularly executives’ psychological orientation [37].

UET points out that the senior managers’ characteristics remarkably influence the environmental behavior of enterprises [38]. The personality characteristics of the CEO are potential variables. Demographic indicators, including but not limited to gender, race, background, and education level [39], are frequently used as proxy variables for CEO characteristics. The application of CEO demographics in empirical research has shown that CEO characteristics can potentially predict a company’s environmental performance [40]. For instance, Ma, Zhang [41] has proved the connection between the education level of senior managers and the disclosure of environmental information in their companies. Executive decision-making is not only influenced by their demographic characteristics, but also by their complex psychological activities. The academic community has extensively explored the influence of common psychological traits such as CEO overconfidence, with most of the focus on the economic and strategic decision-making aspects of the company. Overconfident CEOs tend to be more optimistic about the future and more inclined to mobilize company resources towards challenging entrepreneurial ventures [42], expediting the process of digital transformation [43]. CEO confidence can also affect the adoption of open innovation models in corporations [44], as well as influencing cash holdings and valuations [45]. However, studies have rarely reported whether the CEOs’ psychological biases influence corporate decision to engage in corporate environmental misconducts. Considering that CEOs’ decision is frequently influenced by their sophisticated psychological activities [46], we focused on CEO overconfidence, one of the typical inner personality biases.

Overconfidence is an overly optimistic view of a person’s own knowledge or ability [20,47]. Overconfident CEOs generally possess three distinguishing characteristics. First, they are overly confident in managing and handling difficult situations [20]. They often overestimate their knowledge and ability and simultaneously underestimate the risk of unexpected events [23]. Second, overconfident CEOs tend to have self-attribution biases, where success is attributed to their own faculty while failure is attributed to the external factors [48,49]. Third, overconfident CEOs maintain a positive attitude towards the external environment. They tend to underestimate the adverse impact of unexpected events on the company and naturally believe that their project is more likely to succeed than their competitors [50].

During the recent decades, with the development of behavior theory, studies have loosened the rational assumption of managers and introduced boundary rationality theory into the strategic planning domain of the firm [51]. More and more research has analysed how the irrational behavior of CEOs affects the decision-making process of enterprises, particularly focusing on their propensity [52]. Research on the financial decisions of overconfident CEOs has been relatively in-depth [53]; by contrast, research on the impact of overconfidence on social and environmental decision-making processes is relatively inadequate. The phenomenon of various problems caused by environmental issues is common in various countries, and studying the impact of CEO overconfidence from the perspective of environmental responsibility has significant theoretical and practical significance.

## Hypothesis development

### CEO overconfidence and corporate environmental misconduct

The UET suggests that the characteristics of top managers, such as age, tenure, education level, and some complex psychological variables [38,54], can have an impact on organizational performance, strategy, innovation, and other aspects. Top management is a critical factor influencing and determining corporate environmental initiatives [40,55]. For example, Wei, Ouyang [56] suggested that senior managers shape the values and actions of other organizational members in environmental issues, Mahmoudian and Jermias [57] proved that high CEO salaries may leads to poor corporate environmental performance, and He, Chen [58] found that the senior managers’ characteristics are significant factors affecting the sustainability of enterprises. Therefore, the degree of environmental investment in a company largely depends on the attitude of CEOs toward environmental issues [59], which is highly associated with the personality traits of CEOs [24].

Two main reasons justify the inclination of overconfident CEOs to engage in corporate environmental misconduct. First, overconfident CEOs tend to rely too much on their own decisions and overrate the success rate of their company [60]. Overconfident CEOs, who are prone to being overly optimistic and lacking caution, may underestimate the risks caused by their violations of environmental regulations and believe that the impact of adverse events is limited [61]. Therefore, they have less motivation to engage in environmentally-friendly activities and are rarely conscious that environmental misconduct can cause serious damage [62].

Second, overconfident CEOs are prone to underestimate pressure from stakeholders. Firms satisfy the requirements of internal and external stakeholders by operating in a socially responsible manner [63]. Tang, Qian [31] observed that there is a greater likelihood participate in activities responsible for society among CEOs who rely on stakeholders to obtain greater resource. However, overconfident CEOs are prone to overestimate their capability to control internal management and adapt to understand external context [60]. Even without external assistance, such CEOs believe they have sufficient ability to cope with management risks [31]. Consequently, when trading off between ecological performance and economic profit, overconfident CEOs may have a sense of self-sufficiency and view their companies as being in possession of adequate internal resources and underestimate the pressure from various stakeholders (e.g., environmental authorities, nongovernmental organizations) and finally are involved in environmental misconduct. Accordingly, propose the following assumption:

H1: CEO overconfidence is positively correlated to the engagement of corporate environmental misconducts.

### Contingent effects of governance-level managerial discretion

If CEO overconfidence does affect a firm’s negative environmental activities, which may cause man-made environmental disasters and hurt various stakeholders and ultimately, the shareholders, the contingent factors that may curb this effect have to be identified [31]. According to UET, the level of managerial discretion to some extent determines the impact of managers’ psychological characteristics on corporate behavior [64]. Managerial discretion is interpreted as the scope of decision-making power [65]. The literature on managerial discretion points out that the psychological characteristics of senior managers with more discretion are more embedded in the behavior of the enterprise, thereby affecting the outcomes of the enterprise [66]. It can be speculated that the correlation between CEO overconfidence and corporate behavior is bounded by management discretion [67]. Therefore, our research is concerned with a group of cohesive and closely related corporate governance contingency factors [68]. These factors include shareholder concentration, CEO duality, and board independence, which influence the freedom of managerial discretion and thus moderate the overconfidence–environmental misconduct relationship.

#### Moderating role of shareholder concentration

Agency theory describes the various problems that exist between shareholders and managers [27]. For the benefit of shareholders, more concentrated ownership may exert greater control and more effective supervision on its managers [28]. Compared with dispersed shareholders, large concentrated shareholders supervise managers more efficiently and constrain CEO discretion because of the reduced coordinating cost, thus decreasing the self-seeking behavior of overconfident CEOs [69]. Moreover, Boyd and Solarino [70] also pointed out that major shareholders can utilize formal or informal mechanisms, including voting rights, shareholder activism, and the election or dismissal of board members, to exert pressure on executives. With increasingly prominent environmental issues and relatively tightening regulation, corporate environmental performance has become a vital concern of shareholders. Concentrated shareholding ensures shareholders effectively monitor agents and constrain top executives’ discretion compared with the dispersed shareholders, thus reduces managerial discretion and allows them to promote corporate proactive environmental strategies, weakening the influence of overconfident CEOs on corporate environmental misconduct [69]. By contrast, the more dispersed the equity, the higher the likelihood that shareholders will evade their responsibilities. Such reaction leads to difficulty in effectively monitoring CEO behavior, consequently decreasing the commitment of overconfident CEOs to environmental responsibility. We thus propose the following:

H2: Shareholder concentration weakens the positive relationship between CEO overconfidence and corporate environmental misconduct.

#### Moderating role of CEO duality

The upper echelons theory suggests that the management discretion of top executives can affect their impact on company decisions and outcomes [38,54]. The greater the management discretion of top executives, the greater their impact on the organization [71]. The leadership structure of a board of directors with the same person serving as both the chairman and CEO is known as CEO duality [72]. Corporate governance is often adversely affected by duality [73], because it provides CEOs with greater power and thus greater discretion than usual [74]. When CEO duality exists, board members may fail to govern firms and ensure effective firm performance although they are charged with these responsibilities. Being the chairperson of the board provides the CEO greater discretion to influence board member nominations, board agenda, compensation setting, and so on, which may compromise corporate governance [75]. Duality may confer greater power on the CEO in a relatively unconstrained manner to satisfy his/her own preferences or interests [76].

CEO duality is also a frequently examined in the context of CSR but always as a predictor [77]. There is limited research on the moderating effect of CEO duality. Koch-Bayram and Wernicke [78] found that the negative correlation between CEOs’ military background and fraudulent reporting is enhanced due to the duality of CEOs. Gala and Kashmiri [79] demonstrated that the relationship between CEO overconfidence and corporate risk-taking behavior is positively regulated by CEO duality. CEO duality can persuade or even manipulate the board to evade from taking necessary responsibility and engage in environmental violations. Therefore, managers who concurrently serve as Chairman of the Board of Directors and CEO may have more discretion in allowing overconfidence to push the company in environmentally irresponsible directions. Thus, we propose the following:

H3: CEO duality enhances the positive relationship between CEO overconfidence and corporate environmental misconduct.

#### Moderating role of board independence

The commonly used indicator to measure the monitoring propensity of a board is board independence [39]. Agency theory believes that independent boards can monitor managers effectively through their substantial formal and informal powers, thus reducing agency costs and improving firm performance [29]. Independent directors have played various roles in decision-making, supervision, and consultation on corporate development [80,81].

Stakeholders, including government agencies and the public, have a demand for enterprises to take responsibility for the natural environment because of environmental deterioration. However, top executives may pay less attention to reducing environmental impacts in order to improve financial performance to the maximum extent [82]. In such a scenario, if the proportion of independent directors is high, the CEOs may be forced to engage in environmentally friendly initiatives [83]. Moreover, the establishment of independent directors in enterprises ensures that corporate behavior is aligned, thereby securing the interest of both the shareholders and stakeholders [84]. Independent directors also exert significant impact in monitoring company adherence to social and environmental regulations and guidelines [85].

Therefore, on the one hand, when the proportion of independent directors on the board is higher, effective supervision can be implemented to reduce the managerial discretion of the CEOs, as well as to prevent overconfident CEOs from violating environmental regulations for their own short-term interests [85]. On the other hand, independent directors with expertise in a certain field can provide professional advice on decision-making in environmental protection [86]. Thus, board independence reduces the blind self-confidence of overconfident CEOs and further improves corporate environmental performance. In sum, an independent board is likely to increase its vigilance over the behavior of the overconfident CEO and prevent environmental violations. Thus, we propose the following:

H4: Board independence weakens the positive relationship between CEO overconfidence and corporate environmental misconduct.

## Methodology

### Sample and data sources

We collect data on all Chinese listed firms from 2004 to 2016. The sample data were restricted from 2004 considering that it was the first year of environmental violation events systematically recorded by the Institute of Public and Environmental Affairs (IPE), the number one independent environmental NGO registered in 2006 in China. The IPE is devoted to collect, arrange, and analyse environmental information from companies and governments to establish an environmental performance database. The environmental data of IPE comes from real-time pollution source monitoring and disclosure by local governments in each province and city, along with information that companies are forced or voluntarily disclosing in accordance with relevant legislation and corporate social responsibility requirements. Recent studies have used the IPE data, such as Marquis and Bird [87]. We excluded financial companies with special financial statements, companies receiving special treatment due to abnormal financial conditions, and companies with missing data on key explanatory variables. Ultimately, we obtained 24,828 firm-year observations corresponding to 2,865 unique firms.

The data of corporate environmental misconduct is manually assembled utilizing the environmental violation records disclosed by the IPE. The other data sources come from the China Stock Market and Accounting Research database, which covers most of the financial data of Chinese listed companies [87].

### Measurements

#### Dependent variable: Corporate environmental misconduct [CEI]

Studies on corporate environmental responsibility in emerging economies are limited by data availability [88]. Available databases only contain data from companies in developed countries, such as the Kinder, Leidenberg, and Dow Jones Sustainability Index. Therefore, corporate environmental misconduct measurement is a key issue in the context of China.

As we know, the Environmental Violation Records of Listed Companies disclosed by the IPE is relatively authoritative by adding firms that engaged in environmental conduct to the blacklist in China. It is collected by the IPE from various sources such as the online monitoring system, government supervision, and media reporting on the basis of the environmental violations of firms. For example, as the largest gold producer in China, Zijin mining was recorded once on the List for its toxic water leakage into the river in 2010. We measure corporate environmental misconduct as the frequency of environmental violation records at which a firm appeared on the IPE database each year. Among them, China National Petroleum Corporation (SH.601857) was the most frequently recorded (69 times in 2016) for its pollution discharges exceeding the regulatory standard. Several recent studies have also used this data, such as Marquis and Bird [87].

#### Independent variable: CEO overconfidence

Constructing a plausible measure is the biggest challenge of overconfidence analysis [49]. The psychological orientation of executives is difficult to measure because they may be concerned about social desirability and thus hide their actual personality traits in surveys or interviews [89]. However, managerial decisions or their appearance in the public eye have been used as proxies. Malmendier and Tate [90] suggest that CEOs with excessive confidence generally tend to overrate the company’s future earnings, delay exercising stock options, undertake more mergers and acquisitions, and acquire more shares of company stocks, for example, in China the famous former CEO of COSCO, Jiafu Wei, whose overconfidence led to huge financial losses. Accordingly, in the recent two decades, alternative measures of overconfidence have been proposed, such as mass-media comments on CEOs [20], CEO’s pay relative to other senior managers [91], share and option holdings [92].

Owing to data availability and reliability in China, we use the relative compensation of CEOs as a measure of CEO overconfidence [91] and stockholding change as a robustness test [90]. Generally, the relative compensation of CEOs compared with other top managers reflects their dominant position. The higher the relative compensation, the more important the CEO may feel, and the more overconfident he or she would be. We use the ratio of CEO compensation to the compensation of the top three executives as a representative of CEO overconfidence [91]. To normalize the distribution of the sampled data, we log-transformed the value of relative compensation and lagged for one year.

We also use the shareholding changes of the CEO as a robustness test [51,90]. An executive who did not sell the stocks of the firm when prices rose significantly exhibits overconfidence; a risk-averse CEO considers the translation of stock options into money in the pocket as optimal [52]. Those CEOs who keep or increase their holdings are defined as overconfident CEOs.

#### Moderating variables

Shareholder Concentration. Li, Huang [93] believe that the concentration of shareholders significantly affects the social and environmental behavior of enterprises. It is calculated based on the share percentage of the largest shareholder, expressed as Concentration.

CEO Duality. Duality CEOs confront with fewer constraints and have greater discretion in making decisions, which may result in low environmental performance [76]. Duality is a dummy variable, which is equal to 1 when the CEO serves as the chairman of the board, otherwise equal to 0.

Board independence. The self-serving behavior of CEOs could be monitored by independent outside directors. It is estimated by the excess number of people outside directors relative to directors [68], denoted as INDIR.

#### Control variables

The study brought in a group of control variables, mainly referring to the CEO demographics, enterprise characteristics, and context variables.

First, for the purpose of controlling the influence of CEO characteristics, we introduced several variables [31]: age, gender (1 for male, 0 for female), and education (1 for master’s degree or above, and 0 for other degrees), tenure (the years that a chief executive had been in office), and political connection (1 if the CEO is/was a government official, a representative of the People’s Congress, or a member of the Political Consultative Conference, 0 otherwise, expressed as PC).

Second, we also controlled for other characteristic variables of the company [31], as follows: listing age (the number of years a company has been listed in China’s Stock Exchange, denoted by List Age), enterprise size (the natural logarithm of employees, expressed as Size), ROE (return on equity), firm growth (Operating revenue growth rate from year t -1 to t), leverage (ratio of total liabilities to total assets), slack (current assets/current liabilities ratio), firm risk (the ratio of long-term debt to equity, denoted by Risk), and state ownership (1 for state-owned enterprise and 0 otherwise, denoted as SO).

Third, we accounted for the background variables referred as marketization and competition. We employ the marketization index constructed by Wang, Fan [94] to measure the current development status of regional markets in each province, denoted as MI. Competition is measured logarithmically by subtracting the logarithm of the ratio of CR4 in year t to CR4 in year t-1 from 1, where CR4 represents the market concentration ratio of the top four companies.

Finally, we controlled for industry- or time-relevant factors by incorporating industry- and year-fixed effects.

## Results

### Descriptive statistics and correlation analysis

The mean, standard deviation, and correlation of all variables are listed in Table 1. Table 1 presents that the mean values of CEO Overconfidence, Age, Gender, Education, Tenure, and PC are 0.35, 47.85, 0.94, 0.37, 8.22, and 0.32 respectively, revealing the characteristics of CEOs. The average frequency of corporate environmental misconduct (CEI) is 2.32, indicating that severe environmental violations have occurred among the sampled companies. The correlation coefficients between each variable are all less than 0.4, which is within an acceptable range. In addition, the article also calculated the variance inflation factor (VIF) for them and their cross terms, all of which were below 3. Hence there is no significant multicollinearity between these variables.

**Table 1:** Descriptive statistics and correlations.

	Mean	SD	1	2	3	4	5	6	7	8	9	10	11	12	13	14	15	16	17	18	19	20
1.CEM	2.32	2.35	1.00																			
2.Overconfidence	0.35	0.18	0.03	1.00																		
3.Duality	0.78	0.42	-0.06	-0.06	1.00																	
4.INDIR	0.37	0.05	0.00	0.01	-0.09	1.00																
5.Concentration	36.40	15.44	0.00	-0.06	0.05	0.02	1.00															
6.Tenure	8.22	3.97	-0.11	-0.02	-0.02	0.03	0.04	1.00														
7.Gender	0.94	0.23	-0.01	-0.02	-0.02	-0.05	0.00	0.02	1.00													
8. Education	0.37	0.48	0.02	0.01	-0.03	0.03	-0.02	-0.08	-0.01	1.00												
9. Age	47.85	6.69	-0.01	0.06	-0.17	0.03	0.03	0.08	0.02	-0.08	1.00											
10. List Age	8.54	6.00	-0.15	-0.02	0.19	0.00	-0.11	-0.04	0.02	-0.07	0.09	1.00										
11. Size	21.74	1.34	-0.06	-0.04	0.14	0.04	0.24	0.11	0.03	0.09	0.15	0.26	1.00									
12. Leverage	0.46	0.23	-0.09	-0.05	0.14	-0.03	0.01	-0.05	0.02	-0.05	-0.02	0.35	0.25	1.00								
13. Growth	1.81	213.60	0.00	0.00	0.00	0.00	0.01	0.00	0.00	-0.01	0.00	0.01	0.00	0.00	1.00							
14. ROE	0.06	1.75	0.00	0.01	0.01	0.00	0.01	0.01	0.01	0.00	0.00	-0.01	0.02	-0.01	0.00	1.00						
15. Slack	0.89	12.31	0.01	0.00	-0.02	0.01	-0.01	0.03	0.00	0.01	0.00	0.00	-0.01	-0.07	0.00	0.00	1.00					
16. Risk	61.99	47.48	0.00	0.01	0.04	-0.02	0.06	-0.05	0.01	0.01	0.02	0.06	0.08	0.10	0.00	0.01	-0.02	1.00				
17. Competition	0.51	1.49	0.06	0.05	-0.01	-0.04	-0.01	-0.17	-0.01	-0.04	-0.06	-0.04	-0.09	0.02	-0.03	0.00	-0.07	0.10	1.00			
18. SO	0.46	0.50	-0.10	-0.11	0.25	-0.10	0.22	-0.02	0.08	-0.03	0.05	0.30	0.24	0.24	-0.01	0.00	-0.05	0.06	0.09	1.00		
19. PC	0.32	0.47	0.03	0.03	0.00	-0.02	0.04	-0.04	-0.02	0.04	-0.02	-0.10	0.03	-0.02	0.00	0.01	-0.02	0.01	0.15	-0.01	1.00	
20. MI	7.78	1.83	0.02	0.04	-0.07	0.02	0.02	0.03	0.00	0.05	0.01	-0.08	0.01	-0.09	-0.04	-0.01	0.08	0.10	0.04	-0.13	0.03	1.00

Notes: Correlations above |0.05| were statistically significant at p < .05 (Two-tailed). N = 24,828.

### Hypotheses testing

The article exploits a series of Poisson regression models to prove the assumptions of the previous text, since Poisson model is suitable for dealing with the counting properties of dependent variables. The results of Poisson’s estimation are listed in Table 2. Model 1 only considers control variables, while Model 2 and Model 3 gradually add independent variables (CEO overconfidence) and moderating variables (shareholder concentration, CEO duality, and board independence) on top of Model 1.

**Table 2:** Regression results.

	Dependent variable: CEM			
	Model 1	Model 2	Model 3	Model 4
Overconfidence		0.074(0.047)	0.075(0.046)	-0.054(0.459)
Duality			-0.025(0.336)	-0.025(0.319)
INDIR			-0.207(0.255)	-0.226(0.215)
Concentration			-0.001(0.236)	-0.001(0.287)
Overconfidence× Duality				0.147(0.078)
Overconfidence× INDIR				-1.659(0.056)
Overconfidence× Concentration				0.005(0.052)
Tenure	0.004(0.308)	0.004(0.316)	0.004(0.400)	0.004(0.392)
Gender	-0.057(0.208)	-0.059(0.192)	-0.069(0.129)	-0.067(0.137)
Education	-0.036(0.094)	-0.039(0.074)	-0.030(0.169)	-0.031(0.162)
Age	-0.000(0.761)	-0.001(0.682)	-0.001(0.613)	-0.001(0.646)
List Age	-0.002(0.629)	-0.002(0.515)	-0.002(0.594)	-0.002(0.524)
Size	0.033(0.028)	0.034(0.023)	0.036(0.022)	0.036(0.022)
Leverage	0.168(0.022)	0.161(0.030)	0.123(0.099)	0.120(0.109)
Growth	0.002(0.722)	0.002(0.691)	0.003(0.578)	0.002(0.622)
ROE	0.002(0.727)	0.002(0.761)	0.002(0.650)	0.003(0.620)
Slack	0.045(0.029)	0.043(0.036)	0.037(0.078)	0.037(0.078)
Risk	0.000(0.089)	0.000(0.093)	0.000(0.084)	0.000(0.075)
Competition	0.007(0.767)	0.005(0.849)	0.006(0.824)	0.001(0.972)
SO	-0.139(0.001)	-0.136(0.001)	-0.133(0.001)	-0.130(0.002)
PC	-0.018(0.457)	-0.017(0.490)	-0.009(0.715)	-0.008(0.731)
MI	-0.019(0.026)	-0.020(0.022)	-0.020(0.022)	-0.020(0.019)
Year-fixed effects	YES	YES	YES	YES
Firm-fixed effects	YES	YES	YES	YES
Wald Chi^2^	151.120	46.770	47.53	58.670
N	15,828	13,733	13,645	13,645

Notes: pval in parentheses, two-tailed.

Model 3 in Table 2 shows that CEO overconfidence remarkably and positively affects CEI (β = 0.075, p = 0.046). It reveals that companies with overconfident CEOs more easily participate in environmental misconduct. Thus, hypothesis 1 holds.

Hypothesis 2 suggests that when shareholder concentration is higher, companies with overconfident CEOs rarely behave in environmentally responsible ways. Model 4 reveals that the interaction term between CEO overconfidence and shareholder concentration is statistically remarkable and positive (β = 0.005, p = 0.052), suggesting that shareholder concentration strengths the focal relationship, which is surprisingly contradictory to hypothesis 2.

Regarding Hypothesis 3, the multiplier effect of CEO overconfidence and CEO duality in Model 4 is slightly and significantly positive (β = 0.147, p = 0.078), hereby confirming hypothesis 3.

As to Hypothesis 4, the interaction effect between CEO overconfidence and board independence in Model 4 is significantly negative (β = -1.659, p = 0.056). Therefore, Hypothesis 4 holds. The result reveals that companies with more overconfident CEOs have significantly lower frequency in environmental misconduct with a highly independent board.

We drew an interaction effect graph using standard deviations above and below the average, effectively revealing the moderating effect.

**Fig 1:**
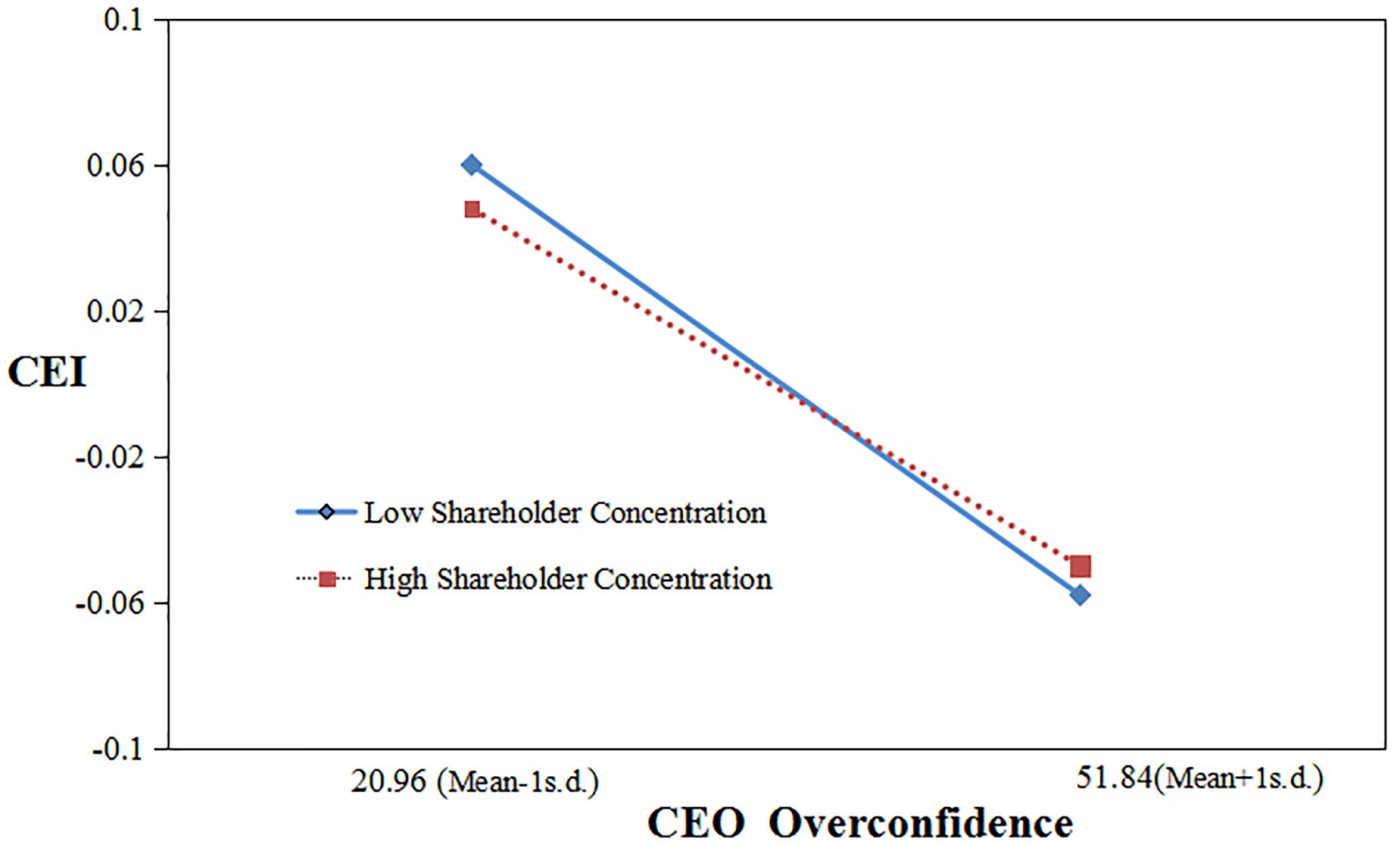
Moderating effect of shareholder concentration.

As presented in Fig 1, at high levels of shareholder concentration, the Overconfidence-CEI relationship is negative; while at low levels of shareholder concentration, the negative connection has been reinforced, indicating that the higher the shareholder concentration, the more likely for overconfident CEOs to engage in environmental violations.

**Fig 2:**
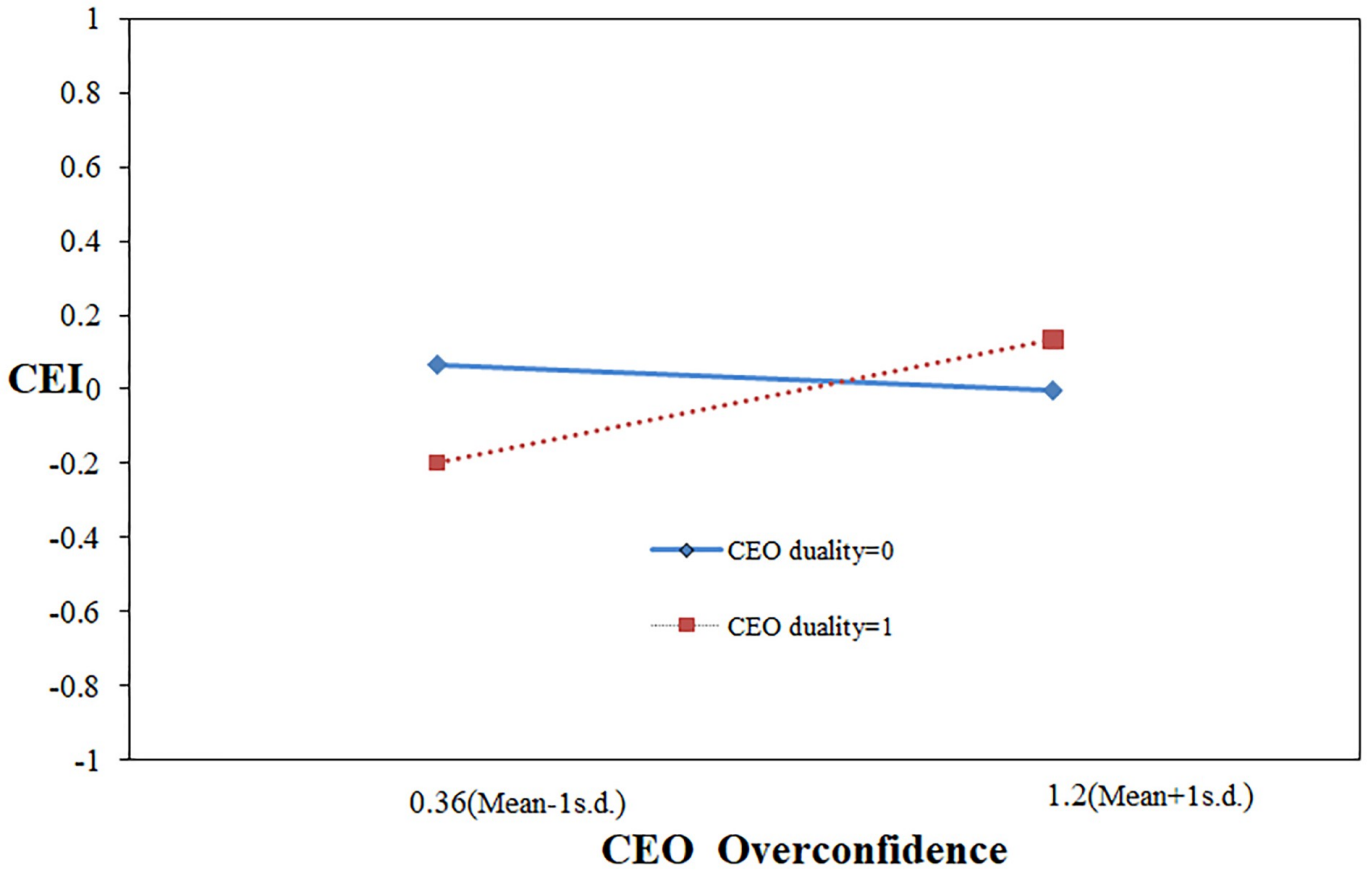
Moderating effect of CEO duality.

Fig 2 shows that when CEO duality presents, the overconfidence-CEI relationship is positive; by contrast, the focal relationship becomes negative with no duality, demonstrating that overconfident CEOs are more prone to be environmentally irresponsible when they are also board chairs.

**Fig 3:**
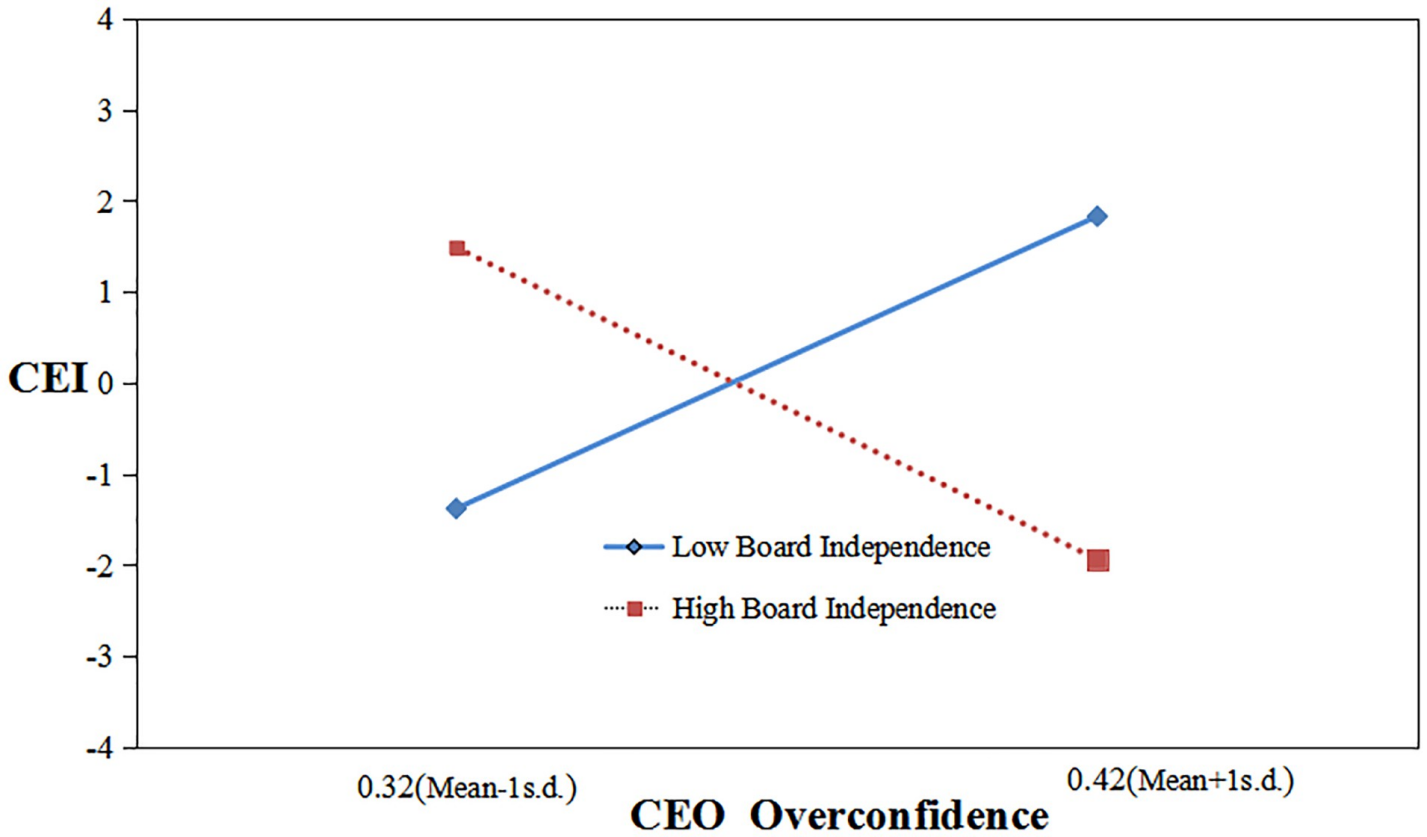
Moderating effect of board independence.

Fig 3 shows that at high levels of board independence, CEO overconfidence negatively affects environmental misconduct; while at low levels of board independence, the opposite is true. This illustrates that overconfident CEOs engage in less environmental violations in firms with higher ratios of independent directors.

### Robustness tests and endogeneity issues

Given the reliability of the research, we propose an ordered categorical variable substitution for CEO overconfidence. According to the relative compensation values of CEO overconfidence, we assign the first fifth of CEOs a value of 1, the last fifth of CEOs a value of -1, and the remaining CEOs which are considered neutral a value of 0 [31]. Then rerun these models using the categorical variable of CEO overconfidence. The robustness results of Model 5, 6, 7, & 8 in Table 3 are in accord with our prior results.

**Table 3:** Robustness tests of categorical salary-based measure of overconfidence.

	Dependent variable: CEM			
	Model 5	Model 6	Model 7	Model 8
Overconfidence		0.021(0.090)	0.022(0.067)	0.034(0.173)
Duality			-0.022(0.385)	-0.021(0.415)
INDIR			-0.203(0.263)	-0.196(0.282)
Concentration			-0.001(0.215)	-0.001(0.230)
Overconfidence× Duality				-0.016(0.578)
Overconfidence× INDIR				-0.493(0.026)
Overconfidence× Concentration				0.002(0.024)
Tenure	0.004(0.308)	0.004(0.322)	0.003(0.416)	0.003(0.414)
Gender	-0.057(0.208)	-0.056(0.213)	-0.065(0.148)	-0.062(0.167)
Education	-0.036(0.094)	-0.038(0.079)	-0.030(0.176)	-0.030(0.176)
Age	-0.000(0.761)	-0.001(0.685)	-0.001(0.619)	-0.001(0.639)
List Age	-0.002(0.629)	-0.002(0.538)	-0.002(0.599)	-0.002(0.582)
Size	0.033(0.028)	0.034(0.023)	0.036(0.022)	0.035(0.022)
Leverage	0.168**(0.022)	0.170(0.021)	0.130(0.080)	0.124(0.097)
Growth	0.002(0.722)	0.002(0.752)	0.002(0.638)	0.002(0.675)
ROE	0.002(0.727)	0.002(0.759)	0.002(0.650)	0.003(0.632)
Slack	0.045(0.029)	0.045(0.029)	0.038(0.068)	0.037(0.072)
Risk	0.000(0.089)	0.000(0.090)	0.000(0.088)	0.000(0.075)
Competition	0.007(0.767)	0.005(0.840)	0.006(0.827)	0.002(0.931)
SO	-0.139(0.001)	-0.137(0.001)	-0.134(0.001)	-0.131(0.001)
PC	-0.018(0.457)	-0.018(0.461)	-0.010(0.682)	-0.010(0.699)
MI	-0.019(0.026)	-0.020(0.022)	-0.020(0.022)	-0.020(0.021)
Year-fixed effects	YES	YES	YES	YES
Firm-fixed effects	YES	YES	YES	YES
Wald Chi^2^	151.120	47.110	47.560	57.590
N	15,828	13,733	13,645	13,645

Notes: pval in parentheses, two-tailed.

We also employ CEO stockholding change to measure overconfidence, 1 for those CEOs that have more stocks of the firm than that of last year, 0 otherwise [90]. The robustness results of Model 9, 10, 11, & 12 in Table 4 are consistent with our previous results, indicating that these assumptions still apply to CEO overconfidence measured using alternative measurement methods.

**Table 4:** Robustness tests of stockholding-based measure of overconfidence.

	Dependent variable: CEM			
	Model 9	Model 10	Model 11	Model 12
Overconfidence		0.063(0.003)	0.065(0.003)	0.065(0.003)
Duality			-0.030(0.241)	-0.032(0.216)
INDIR			-0.200(0.270)	-0.222(0.223)
Concentration			-0.001(0.194)	-0.001(0.277)
Overconfidence× Duality				0.100(0.028)
Overconfidence× INDIR				-1.412(0.091)
Overconfidence× Concentration				0.005(0.044)
Tenure	0.004(0.308)	0.005(0.246)	0.004(0.246)	0.004(0.324)
Gender	-0.057(0.208)	-0.056(0.211)	-0.066(0.143)	-0.067(0.137)
Education	-0.036(0.094)	-0.034(0.120)	-0.025(0.249)	-0.028(0.196)
Age	-0.000(0.761)	-0.000(0.848)	-0.000(0.739)	-0.001(0.680)
List Age	-0.002(0.629)	-0.001(0.684)	-0.001(0.741)	-0.002(0.566)
Size	0.033(0.028)	0.031(0.036)	0.033(0.032)	0.035(0.026)
Leverage	0.168(0.022)	0.168(0.022)	0.127(0.087)	0.118(0.114)
Growth	0.002(0.722)	0.002(0.726)	0.002(0.617)	0.002(0.627)
ROE	0.002(0.727)	0.002(0.746)	0.003(0.632)	0.003(0.641)
Slack	0.045(0.029)	0.044(0.032)	0.037(0.076)	0.035(0.090)
Risk	0.000(0.089)	0.000(0.092)	0.000(0.091)	0.000(0.080)
Competition	0.007(0.767)	0.008(0.748)	0.009(0.731)	0.002(0.953)
SO	-0.139(0.001)	-0.138(0.001)	-0.134(0.001)	-0.128(0.002)
PC	-0.018(0.457)	-0.018(0.452)	-0.010(0.679)	-0.009(0.728)
MI	-0.019(0.026)	-0.019(0.030)	-0.019(0.030)	-0.020(0.021)
Year-fixed effects	YES	YES	YES	YES
Firm-fixed effects	YES	YES	YES	YES
Wald Chi^2^	151.12	52.93	53.35	67.06
N	15,828	13,733	13,645	13603

Notes: pval in parentheses, two-tailed.

To address for the endogeneity concerns, the study corrects sample selection bias utilizing the Heckman two-stage method [95]. The ‘inverse mill ratio (IMR)’ is obtained in the first step of regression, which was then brought into all models. The Poisson estimates for the above steps are shown in the Table 5. In Panel A, we adopted the ‘Probit Model’ for the first step of regression, with the observations of China’s listed companies from 2004 to 2016 where the dependent variable was ‘CEI Dummy’, which equals 1 if the firm was appeared on the Environmental Violation Records of Listed Companies disclosed by the IPE, and 0 otherwise. The independent variables included are: the logarithm of the number of employees, Cash, INDIR, and the proportion of state ownership.

**Table 5:** Results for endogeneity checks with Heckman two-stage procedure.

Panel A: the First-step regression—model employed to estimate inverse Mills									
Variable	Size	Cash	INDIR	State Share	Year	Cons	N	Pseudo R^2^	LR chi^2^
CEM Dummy	0.000	0.000	0.256	0.024	YES	1.017	24637	0	4.19
	(0.671)	(0.364)	(0.174)	(0.235)		(0.000)			
Panel B: the second-step regression—after introducing inverse Mills									
			**Dependent variable: CEM**						
			Model 14		Model 15	Model 16		Model 17	
Overconfidence					0.228(0.041)	0.229(0.043)		-0.063(0.816)	
Duality						-0.100(0.100)		-0.108(0.079)	
INDIR						-0.414(0.418)		-0.434(0.396)	
Concentration						0.001(0.653)		0.001(0.645)	
Overconfidence× Duality								0.341(0.237)	
Overconfidence× INDIR								-3.580(0.205)	
Overconfidence× Concentration								0.016(0.013)	
Tenure					-0.056(0.000)	-0.056(0.000)		-0.056(0.000)	
Gender			0.236(0.101)		-0.071(0.464)	-0.079(0.415)		-0.075(0.444)	
Education			-0.835(0.000)		-0.031(0.475)	-0.036(0.421)		-0.036(0.412)	
Age			0.022(0.000)		0.008(0.013)	0.007(0.036)		0.007(0.033)	
List Age			-0.030(0.000)		-0.061(0.000)	-0.060(0.000)		-0.060(0.000)	
Size			0.329(0.000)		0.019(0.220)	0.019(0.241)		0.019(0.246)	
Leverage			-0.254(0.253)		-0.050(0.667)	-0.044(0.705)		-0.047(0.686)	
Growth			0.026(0.238)		0.003(0.779)	0.003(0.810)		0.003(0.805)	
ROE			0.014(0.630)		0.005(0.502)	0.005(0.489)		0.006(0.428)	
Slack			0.116(0.059)		0.100(0.005)	0.099(0.006)		0.098(0.006)	
Risk			-0.004(0.000)		0.001(0.004)	0.001(0.004)		0.001(0.005)	
Competition			-0.185(0.000)		0.026(0.213)	0.026(0.216)		0.024(0.248)	
SO			0.333(0.000)		-0.279(0.000)	-0.291(0.000)		-0.291(0.000)	
PC			0.065(0.331)		0.004(0.920)	0.005(0.911)		0.007(0.881)	
MI			0.104(0.000)		-0.006(0.588)	-0.007(0.527)		-0.007(0.498)	
IMR			-17.842(0.001)		3.951(0.236)	1.200(0.776)		0.952(0.820)	
Cons_			3.676(0.019)		1.601(0.101)	2.562(0.049)		2.741(0.035)	
Year-fixed effects			YES		YES	YES		YES	
Firm-fixed effects			YES		YES	YES		YES	
Adjusted R^2^			0.032		0.044	0.044		0.044	
N			15,789		13,701	13,701		13,701	

Notes: pval in parentheses, Two-tailed.

Panel B in Table 5 suggests that the coefficients of IMRs is not significant. Thus, our model is not plagued by sample selection bias. After ushering in IMR for controlling sample selection bias, all results in Panel B of Table 5 still hold true. The results of Model 15, 16, and 17 in panel B are in accord with Model 2, 3, and 4 of Table 2, where CEO overconfidence is still positively correlated with corporate environmental misconduct, and the moderating effects of governance-level managerial discretions are in accord with those in Table 2.

## Discussion and conclusion

### General discussion

The research for this article examined that how CEO overconfidence can lead to corporate environmental misconduct in China. On account of UET [38], it’s posited that when senior managers become overly confident, their companies take a series of actions that are environmentally illegal. This study also explored how management discretion moderates the impact of overconfidence on environmental misconduct. An empirical analysis was conducted on a sample of Chinese quoted companies from 2004 to 2016. The primary research results as follow:

First, the findings illustrate that improper environment behavior in the corporate can be positively motivated by CEO overconfidence, confirming the hypothesis that companies with overconfident CEOs prefer implementing environmental violation. When making environmentally relevant decisions, the CEOs with excessive confidence easily exhibit blind optimism than their peers without overconfidence. Overconfident CEOs felt that the impact of adverse events was limited and underestimated the risk posed by violations of environmental regulations. Under this circumstance, the CEO has motivation to ignore stakeholder interests and pressure and lead to environmental violations [31,85].

Second, the empirical results emphasize the moderating effect of governance-level managerial discretion, either strengthening or weakening the overconfidence–environmental misconduct relationship. The results demonstrate that CEO duality empowers CEOs with greater decision-making power and enhances the positive impact of CEO overconfidence on corporate environmental misconduct. With more independent directors in the board, the power and discretion of overconfident CEOs are restricted, thereby reducing the irresponsible activities that affect the environment [96].

However, the assumed negative moderating effect of shareholder concentration doesn’t hold true: the correlation coefficient between overconfidence and concentration is significantly positive (β = 0.005, p = 0.052). This relationship indicates that the more concentrated the shareholders, the higher is the likelihood that overconfident CEOs would engage their firms in environmental violations in China. The intuitive assumption of this result is that corporate environmental performance can be said to be a private provision of a public good [97]. Simultaneously, high costs are often required to be environmentally friendly and to comply with environmental law. Thus, the public good nature of environmental performance and the cost-benefit analysis inhibits the motivation of larger stakeholders’ motivation in monitoring overconfident CEOs’ environmental behavior, resulting in a lower likelihood of the company considering environmental measures as shareholders become more concentrated [97].

### Theoretical contributions

The research has made certain contributions at the theoretical level to the literature concerned with UET, CSR, and emerging economies. First, this research extends UET by investigating the influence of psychological biases of top executives in the ecological setting. Prior research has primarily focused on that how observable management characteristics (such as gender, age, background) affect economic outcomes [66]; we explore that how unobservable CEO overconfidence affect ecological outcomes. Although confident leaders have usually been valued as competent, overconfidence may be detrimental to corporate ecological outcomes. Moreover, we find that several managerial discretion factors at the governance level, such as shareholder concentration, CEO duality, and board independence can curb or spur corporate environmental violations caused by CEO overconfidence, which broadens our understanding of the UET in the ecological setting.

Second, we contribute to CSR research in that although researchers and managers have exerted considerable efforts to understand CSR, the opposite-termed corporate social irresponsibility is less known [98] and much less, specific environmental misconduct [99]. Whereas the current literature explores corporate environmental behavior from institutional theory [100], stakeholder theory [101], and resource-based perspectives [102], our research attaches close attention to the psychological biases of the CEO within the context of UET—that is, the idea that the CEOs with excessive confidence easily participate in environmentally improper behavior compared with other CEOs, which enriches the CSR literature.

Third, the research is also beneficial for literature involved in the growth of emerging economies. Our research adopts a large vertical dataset from multiple industries in the context of China. Previous studies on psychological bias and corporate environmental misconduct have mainly focused on Western developed countries, whereas reports on transitional economies such as China are inadequate [93]. Compared to individualistic backgrounds, the impact of overconfidence and managerial discretion in collective social environments is arguably weaker [103]. Another difference is that China is experiencing rapid growth while also experiencing severe environmental damage, whereas the environmental issues of Western countries is not as severe. Furthermore, unlike developed countries, Chinese institutional background has been criticized for its failure in enterprise environmental and social responsibility matters [104]. In sum, with the conspicuous disparity between collective and individualistic societies, developed and underdeveloped countries, as well as Western and Eastern countries [105], a narrow focus on developed nations restricts the theoretical completeness of the field. This study has certain reference value for preventing corporate environmental misconduct in other emerging economies which are also confronted with serious environmental misconduct and lacking sound regulation [3]. Our research thus devotes to the UET and CSR literature through encapsulating and elucidating CEO overconfidence, managerial discretion, and corporate environmental misconduct in a novel context.

### Managerial implications

The research has significant managerial implications. The personal psychological biases of CEOs significantly affect corporate environmental misconduct, as explained by UET. Our findings indicate that firms run by overconfident CEOs may cause natural disasters. Thus, when recruiting for senior positions, enterprises should make a profound investigation into the psychological biases of managers and clarify the potential impact of these biases on CSR.

Considering corporate governance perspective on managerial discretion, board members and shareholders should implement an effective governance mechanism and intimately supervise agency problems that may cause environmental violations. Our findings believe that board independence may alleviate the influence of overconfident CEOs in taking irresponsible initiatives harmful to the environment, which should be further emphasized. However, the presence of shareholder concentration and CEO duality would render firms with overconfident CEOs engaged in more environmental violations, suggesting that firms should be aware of the drawbacks of such governance mechanisms in the ecological aspect.

### Limitations and future research directions

Our research is limited and worth further exploration. First, although the Environmental Violation Records of Listed Companies disclosed by the IPE is authoritative, the precise environmental performance or violation data remain unavailable, which is a major limitation of this study. Further studies should employ enhanced corporate environmental misconduct measurements to explore its antecedents and consequences. Moreover, this study does not cover non-listed firms or small and medium-size enterprises whose environmentally irresponsible behavior is pervasive. Therefore, if there is more available data in the future to test the robustness of research results, our research scope may be further expanded.

Second, though objective data are accepted as proxies of overconfidence, most papers use a variety of measures to proxy overconfidence. However, we only used relative compensation and shareholding change as measures due to data limitation in China. Further studies should use other measures to more clearly reflect the concept of overconfidence.

Third, in addition to managerial discretion factors at the governance level, the relationship between overconfident CEOs and corporate environmental misconduct may also be moderated by other characteristics of senior managers, other organizational factors, and external business environment. These factors may play a moderating or mediating role in this relationship, providing opportunities for future research.
